# The ‘Where, What, How and Who’ of Head Accelerations in Rugby Union. Head Acceleration Events From Men's and Women's Northern and Southern Hemisphere Competitions

**DOI:** 10.1002/ejsc.12295

**Published:** 2025-05-22

**Authors:** Gregory Roe, Thomas Sawczuk, James Tooby, Cameron Owen, Lindsay Starling, Ross Tucker, Keith Stokes, James Brown, Matt Cross, Éanna Falvey, Sharief Hendricks, Simon Kemp, Clint Readhead, Karen Rasmussen, Danielle Salmon, Ben Jones

**Affiliations:** ^1^ Carnegie Applied Rugby Research (CARR) Centre Carnegie School of Sport Leeds Beckett University Leeds UK; ^2^ England Performance Unit Rugby Football League Manchester UK; ^3^ World Rugby Dublin Ireland; ^4^ Institute of Sport and Exercise Medicine (ISEM) Department of Exercise University of Stellenbosch Stellenbosch South Africa; ^5^ Rugby Football Union Twickenham UK; ^6^ Centre for Health and Injury and Illness Prevention in Sport University of Bath Bath UK; ^7^ UK Collaborating Centre on Injury and Illness Prevention in Sport (UKCCIIS) University of Bath Bath UK; ^8^ Premiership Rugby London UK; ^9^ School of Medicine & Health University College Cork Cork Ireland; ^10^ Division of Physiological Sciences Department of Human Biology Faculty of Health Sciences University of Cape Town Cape Town South Africa; ^11^ London School of Hygiene and Tropical Medicine London UK; ^12^ South Africa Rugby Union Cape Town South Africa; ^13^ New Zealand Rugby Union People Safety & Wellbeing Wellington New Zealand; ^14^ School of Behavioural and Health Sciences Faculty of Health Sciences Australian Catholic University Brisbane Australia

**Keywords:** health, injury and prevention, team sport

## Abstract

This study aimed to quantify and compare mean head acceleration event (HAE) incidence within and between men's and women's rugby union competitions; quantify the incidence of HAEs during all contact‐events and describe individual player incidence. Players competing during the 2022/2023 season in women's (337 players; Premiership Women's Rugby, Farah Palmer Cup) and men's (371 players; Premiership Rugby, Currie Cup and Super Rugby) competitions wore instrumented mouthguards (iMGs). Mean HAE incidences using peak linear (PLA) and peak angular acceleration (PAA) were quantified by sex, positional groups and individual players per competition and for contact‐events across a range of magnitude thresholds. Within positional groups, there was high between‐player variability, with some players experiencing up to a 3‐fold greater mean HAE incidence than their positional average. Per full‐game equivalent (FGE), men had significantly higher HAE incidences in most positional groups and HAE magnitude thresholds compared to women ranging from approximately 0.11–3.44 HAEs per FGE. Incidence of HAEs (PLA > 25 g) per FGE was lowest in scrums (0.00–0.04/FGE) and highest for tackles and ball carries (0.21–1.97/FGE) in both women and men, whereas mauling was a frequent source of HAEs for men's back row (0.95/FGE). No significant differences were observed between competitions for most positional groups and HAE magnitude thresholds in both men and women. Per FGE, HAE incidences were similar within, but significant differences were apparent between men's and women's players. The scrum had the lowest HAE incidence of all contact‐events. Individual players can show large variation from the mean, emphasising the importance of HAE mitigation strategies that include individual player monitoring and management processes.

**Summary**

- Within positional groups, there was high between‐player variability in both men and women players, with some players experiencing up to three times greater mean HAE incidence than their positional average, emphasising the importance of HAE mitigation strategies that prioritise individual player monitoring processes and management strategies.
- Future research should prioritise investigating the factors that contribute to between‐player variability to further inform HAE mitigation strategies.
- On average, men's players had a significantly higher HAE incidence per FGE in most positional groups and HAE magnitude thresholds when compared to women's players, whereas no significant differences were observed between competitions for the majority of playing positions and HAE magnitude thresholds per FGE in both men and women.
- In addition to the tackle and ruck, mauls may significantly contribute to HAE exposure in men's back row, whereas the incidence of HAEs in scrums and lineouts is of less concern for both men's and women's players.

## 1 Introduction

Rugby union is a contact sport played by both men and women globally (Heyward et al. 2022). There is a concern in rugby union regarding the potential long‐term effects of repetitive head impacts on brain health (Iverson et al. 2023). Research in American football has suggested significant associations between retrospective estimations of cumulative head impacts throughout players' careers and chronic traumatic encephalopathy (Daneshvar et al. 2023) and neurocognitive impairments in later life (Montenigro et al. 2017). Thus, accurately quantifying player head impacts and investigating their clinical relevance in contact sports are of utmost importance (Tooby et al. 2024).

Instrumented mouthguards (iMGs) have recently been used to quantify head impacts during training and competition (Bussey et al. 2023; Roe, Sawczuk, Owen, et al. 2024; Tooby et al. 2023). Instrumented mouthguards are a validated technology for measuring head acceleration events (Jones et al. 2022): the acute acceleration of the head in response to an impacting force to the head or body (Kuo et al. 2022). To date, findings in both men and women's rugby union, based on either data from one competition (e.g., Bussey et al. 2024) or aggregated across competitions (e.g., Roe, Sawczuk, Owen, et al. 2024; Tooby et al. 2023), suggest that on average, the majority of HAEs (∼95%–98%) experienced by players are lower in magnitude (i.e., < 30 g or 2000 rad/s^2^). However, it is not known if the number of HAEs experienced by players differs between competitions. Furthermore, the extent to which HAE incidence differs between men's and women's competition has only been assessed in a small sample aggregate of multiple competitions (Tooby et al. 2023) and warrants further investigation. Additionally, HAE incidence has yet to be quantified for contact‐events other than the tackle and ruck (e.g., maul and scrum). Such information is important for policymakers to determine appropriate HAE mitigation strategies.

The HAE incidence in rugby union has primarily been reported as mean findings, with little attention given to the between‐player variation (Bussey et al. 2023; Tooby et al. 2023). Although a mean can provide a useful summary of important data, as the variance around the mean increases, the summary becomes less adequate (Speelman and McGann 2013). This could be a particular issue within Poisson distributed analyses, such as those used in HAE incidence studies (e.g., Roe, Sawczuk, Owen, et al. (2024)), where the mean is equal to the variance. Therefore, as the mean increases, so too does the variance. As such, it is unclear if some individual players are disproportionately accumulating more HAEs, which is important given the potential effects of long‐term HAE exposure on brain health (Daneshvar et al., 2023). In view of this, player‐monitoring strategies can only be optimised if we understand whether HAE incidence varies between players (Svaldi et al. 2020). Furthermore, understanding between‐player variability may help determine future directions of research to support HAE mitigation strategies (e.g., identifying factors that may influence a disproportionate accumulation of HAEs in certain individuals). Therefore, the aims of the present study were to quantify and compare the mean HAE incidence of players in and between different men's and women's rugby union competitions by the positional group; to quantify the incidence of HAEs across all contact events and to explore how incidence varies between players in the same position.

## 2 Methods

### 2.1 Study Design

A prospective observational study was conducted in rugby union players from three professional men's and two semi‐professional women's rugby union competitions who participated in a World Rugby deployment of iMGs during the 2022/23 season. These included the highest level of domestic women's competition in England (Premiership Women's Rugby) and New Zealand (Farah Palmer Cup), and the highest level of domestic men's competition in England (Premiership Rugby), South Africa (Currie Cup) and Australia and New Zealand (Super Rugby). Considering the specific position played in each match (some players played different positions in different matches), players were clustered into the following positional groups (Quarrie et al. 2013); front‐five, back‐row, half‐backs, centres and outside‐backs players. The number of players and player‐matches for each competition and positional group are presented in Table 1. Ethics approval was received from the University Ethics Committee (REF: 108638).

**TABLE 1:** The number of players (player‐matches) per competition by the positional group.

	Front five	Back row	Half backs	Centres	Outside backs
Premiership Women's Rugby (W)	39 (143)	30 (86)	15 (40)	21 (67)	26 (67)
Farah Palmer Cup (W)	102 (251)	48 (111)	31 (76)	38 (71)	50 (116)
Premiership Rugby (M)	80 (429)	41 (206)	19 (69)	18 (99)	35 (177)
Currie Cup (M)	53 (230)	38 (164)	25 (83)	19 (72)	27 (94)
Super Rugby (M)	30 (78)	10 (21)	5 (13)	8 (17)	13 (33)

Abbreviation: M, men; W and women.

### 2.2 Instrumented Mouthguard Data

All players underwent 3D dental scans and were provided with custom‐fit iMGs (Prevent Biometrics, Minneapolis, MN, USA). The iMG contained an accelerometer and gyroscope that sampled at 3200 Hz with measured ranges of ± 200 g and ± 35 rad/s. Coupling of the iMG to the upper dentation was determined by way of infrared proximity sensors. Laboratory validation of the Prevent Biometrics iMG yielded a concordance correlation coefficient of 0.984 (95% CI: 0.977–0.989), whereas field‐based video‐verification analysis yielded a positive predictive value of 0.94 (0.92–0.95) and a sensitivity value 0.75 (0.67–0.83) during the on‐field video‐verification validation (Jones et al. 2022).

A discretised period of kinematics (−10 ms and +40 ms from trigger point) was stored for each HAE and linear kinematics were transformed to the estimated head centre of gravity (CoG) using the relative acceleration equation. Each HAE was classified as a true positive or false positive by an in‐house Prevent Biometrics algorithm based on infrared proximity sensor readings and kinematics. Linear and angular kinematics were filtered by Prevent Biometrics using a four‐pole, zero phase and low‐pass Butterworth filter with a 200 Hz cutoff frequency. Another in‐house Prevent Biometrics algorithm classified HAEs based on the level of noise in the signal as minimal (*n* = 61,333), moderate (*n* = 4714) or severe (*n* = 1641). Additional filtering was applied via Prevent Biometrics to HAEs classified with moderate and severe noise with cutoff frequencies of 100 and 50 Hz, respectively. Peak linear acceleration (PLA) and peak angular acceleration (PAA) values were calculated by extracting peak resultant values from each HAE.

Video analysis data of all contact‐events during all competitions were acquired from Opta, provided by StatPerform (Chicago, IL, USA). Instrumented players' data were exported from the Prevent Biometrics Portal (Prevent Biometrics, Minneapolis, MN, USA), and PLA and PAA values below 5 g and 400 rad/s^2^, respectively, were excluded at this point based on previous recommendations (Tooby et al. 2023). Accelerometer, gyroscope and proximity sensor data were synchronised to video timestamps of contact‐events using MATLAB (MathWorks, UK, version R2023a). A HAE was linked to a collision event if their timestamps occurred within 10 seconds of one another. Only contact‐events that had corresponding proximity sensor data for the instrumented player were used in the analysis (Tooby et al. 2023). This method had an 86.4% accuracy (unpublished data). Subsequently, video verification of all HAEs was undertaken to ensure they were associated with a contact‐event. Contact‐events that had proximity sensor data for the instrumented player were used in the analysis (Tooby et al. 2023).

### 2.3 Head Acceleration Event Thresholds

The linear trigger threshold utilised using iMGs can cause a ‘linear trigger bias’ that can result in false negatives (Tooby et al. 2024; Wang et al. 2021). These false negatives have been shown to occur in PLA magnitudes up to 30 g in iMGs with a 10 g trigger threshold (Wang et al. 2021). In the present study, the iMG technology utilised an 8 g trigger threshold. Therefore, modelling of count data included all HAEs, in addition to HAEs at magnitudes > 25 g to minimise the risk of false negatives, increasing in 15 g increments. A corresponding PAA magnitude of > 2 krad/s^2^ was used, increasing in increments of 1 krad/s^2^ (Roe, Sawczuk, Owen, et al. 2024).

#### 2.3.1 Statistical Analysis

To quantify the HAEs experienced by players during match‐play, generalised linear mixed models, assuming a Poisson distribution, were used. Models were run individually for counts of all HAEs and of PLA > 25, 40 and 55 g, PAA > 2, 3 and 4 krad/s^2^ and combinations of PLA > 25 g and PAA > 2 krad/s^2^, PLA > 40 g and PAA > 3 krad/s^2^ and PLA > 55 g and PAA > 4 krad/s^2^. Attempts were made to model higher magnitudes, but the models would not converge beyond those detailed here. All analyses were conducted in R (4.3.2), with the glmmTMB (Brooks et al. 2024) and emmeans (lenth et al. 2024) packages used.

### 2.4 Competition and Positional Group Mean HAE Incidence

For mean incidences, two models were produced for each magnitude threshold; one to compare differences between competitions and one to compare differences between sexes for each positional group. In both models, a fully factorial fixed effects structure was used. For the competition analysis, positional group, competition and the logarithm of minutes played were used to estimate the number of HAEs experienced by an athlete for each positional group competition combination per full game equivalent (FGE; i.e., 80 min). At the time of writing, World Rugby guidelines state that transgender women cannot play women's rugby. Therefore, it was assumed that women's players were all female and men's players were all male. For the sex analysis, positional group, sex and the logarithm of minutes played were used to estimate the number of HAEs experienced by an athlete for each positional group sex comparison per FGE. In both models, random effects of player ID (to account for players competing in multiple matches) and fixture ID (to account for multiple players playing in the same match) were added as individual random intercepts. Incidences are presented as mean and 95% confidence intervals (CIs) to two decimal places. Differences between means were interpreted as significant and meaningful when the confidence intervals of the estimates did not overlap (Noguchi and Marmolejo‐Ramos 2016).

### 2.5 Contact‐Event HAE Incidence

Unmodelled estimates of the incidence of HAEs at different magnitudes for each contact‐event were produced using the *epiR* package (Stevenson and Sergeant, 2024). Ninety‐five percent confidence intervals were calculated using the “byar” methodology (Rothman, 2012). HAE incidences are provided per FGE based on the contact‐event frequencies provided by Roe, Sawczuk, Collins, et al. 2024.

### 2.6 Individual Player HAE Incidence

For individual incidences, model estimated means from the 25 g model were used. To obtain individual means, the competition, positional group, median minutes for the player and player ID random effect were used. This differs from the positional group means, which used the competition, positional group and competition*positional group median minutes played, with no random effect adjustment. The random effect adjustment for the player adjusts the player's mean incidence up or down dependent on the quantity and magnitude of data supporting its movement.

## 3 Results

### 3.1 HAE Incidence in Women's and Men's Competitions

**FIGURE 1:**
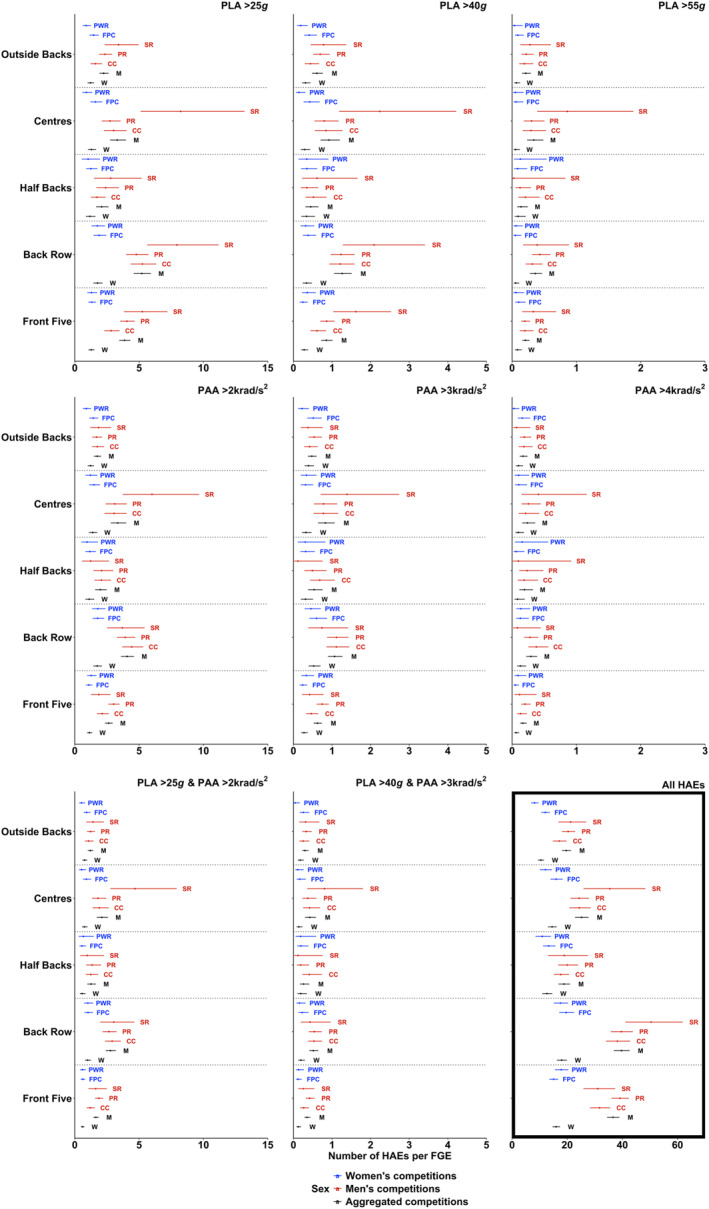
The mean HAE incidence for different HAE magnitude thresholds experienced by men's and women's positional groups per FGE in different competitions and aggregated by sex. The model for PLA > 55 g and PAA > 4 krad/s^2^ did not converge. Cumulative HAE incidence is presented in the bottom right panel. Abbreviation: CC, Currie Cup; FPC, Farah Palmer Cup; M, men's; PR, Premiership Rugby; PWR, Premiership Women's Rugby; SR, Super Rugby W, women's.

HAE incidence was not significantly different between women's competitions in any positional group for any HAE magnitude threshold (Figure 1), except for all HAEs for outside backs (Farah Palmer Cup 12.0 [10.6–13.7] vs. Premiership Women's Rugby 8.1 [6.9–9.6]). Similarly, differences between men's competitions were predominantly not significant (Figure 1). Where significant differences were observed, certain positional groups in Super Rugby (i.e., front five, centres and outside backs) demonstrated higher HAE incidences than for those positions in other competitions, with HAE incidence ranging between approximately 1.5–5.4 HAEs per FGE on average.

### 3.2 Differences Between Women's and Men's Competitions

HAE incidence was significantly lower in women than in men for all PLA magnitudes, except for halfbacks, where women's HAE incidence was lower for PLA > 25 g only (Figure 1). Men versus Women differences ranged from approximately 6.2 to 21.8 for all HAEs and 0.11 to 3.44 HAE per FGE at modelled thresholds, with back row, front five and centres demonstrating the largest differences. Both PAA and the combination of PAA and PLA magnitude thresholds showed similar patterns, where women had significantly fewer HAE than men at lower HAE thresholds (PAA > 2 krad/s^2^; PLA > 25 g and PAA > 2 krad/s^2^), with values ranging from approximately 0.5–2.5 HAE per FGE. No differences were observed at the highest HAE thresholds modelled (PAA > 4 krad/s^2^; PLA > 55 g and PAA > 4 krad/s^2^). At PAA > 3 krad/s^2^ and PLA > 40 g and PAA > 3 krad/s^2^, women's front five, back row and centres had lower HAE incidence than men, all < 0.35 per FGE.

### 3.3 HAE Incidence in Contact Events

**FIGURE 2:**
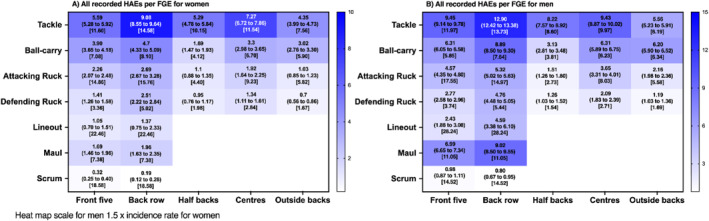
Incidence (95% CI) of all HAEs per FGE associated with each contact‐event for women and men rugby union players per positional group [median contact events per FGE].

**FIGURE 3:**
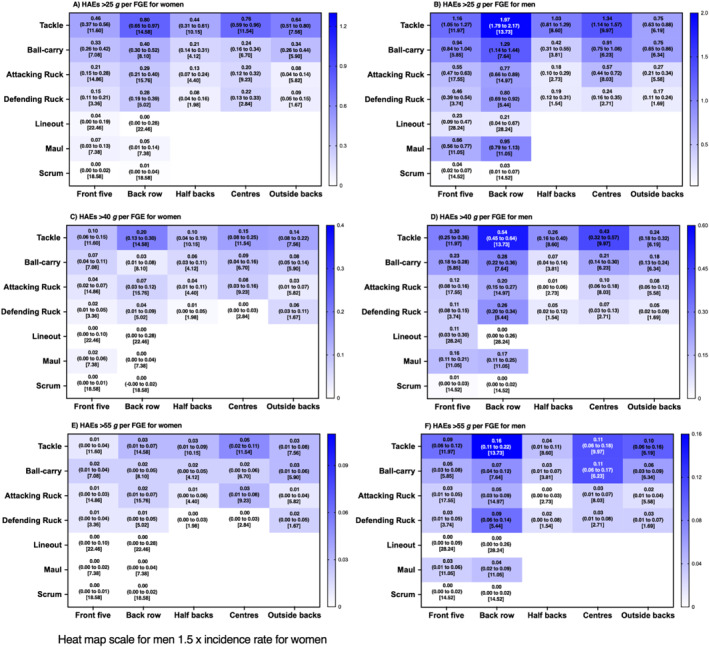
Incidence (95% CI) of HAEs above PLA thresholds per FGE associated with each contact‐event for women and men rugby union players per positional group [median contact events per FGE].

**FIGURE 4:**
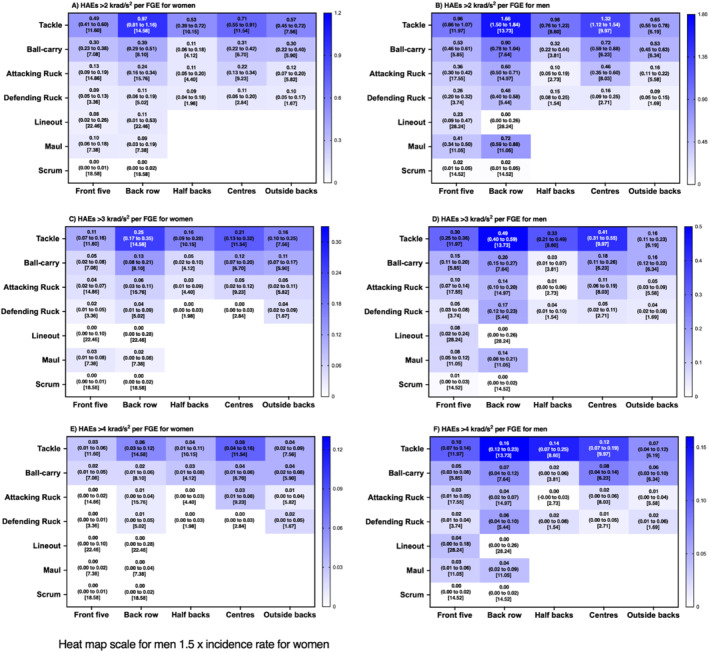
Incidence (95% CI) of HAEs above PAA thresholds per FGE associated with each contact‐event for women and men rugby union players per positional group [median contact events per FGE].

**FIGURE 5:**
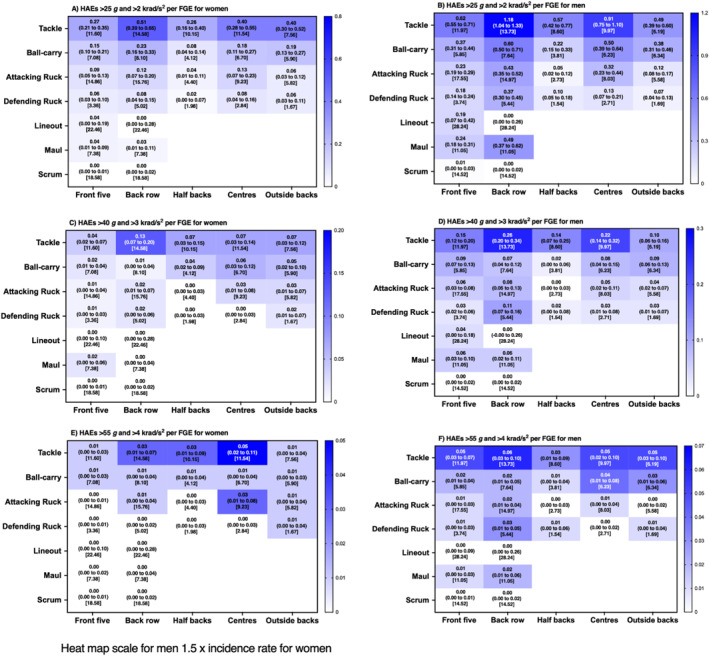
Incidence (95% CI) of HAEs above combined PLA and PAA thresholds per FGE associated with each contact‐event for women and men rugby union players per positional group [median contact events per FGE].

Figures 2, 3, 4, 5 detail the incidence of HAEs associated with each contact‐event per FGE. Incidence rates decreased as HAE magnitude thresholds increased for both men's and women's players across all positions. For all HAEs (PLA > 5 g or PAA > 400 rad/s^2^), contact‐event incidence rates ranged from 0.19 (0.12–0.28 per FGE; scrum in women's back row) to 12.90 (12.42–13.38 per FGE; tackle in men's back row) per FGE.

At the lower magnitude thresholds (PLA > 25 g, Figure 3A,B; PAA > 2000 rad/s^2^, Figure 4A,B; and a combination of both; Figure 5A,B), incidence rates ranged from 0.00 (0.00–0.02 per FGE; scrum in women's front five; Figure 3A) to 1.97 (1.79–2.17 per FGE; tackling in men's back row; Figure 3B) per FGE. Above PLA of 40 g (Figure 3C,D; PAA > 3000 rad/s^2^, Figure 4C,D; and a combination of both Figure 5C,D), incidence rates ranged from 0.00 (0.00–0.01 per FGE; scrums in women's front five) to 0.54 (0.45–0.64 per FGE; tackle in men's back row) per FGE. At the highest thresholds analysed (PLA > 55 g, Figure 3E,F; PAA > 4000 rad/s^2^, Figure 4E,F; and a combination of both, Figure 5E,F), incidence rates ranged from 0.00 (0.00–0.01 per FGE; scrums in women's front five) to 0.16 (0.11–0.22 per FGE; tackle in men's back row) per FGE.

### 3.4 Individual Player HAE Incidence

**FIGURE 6:**
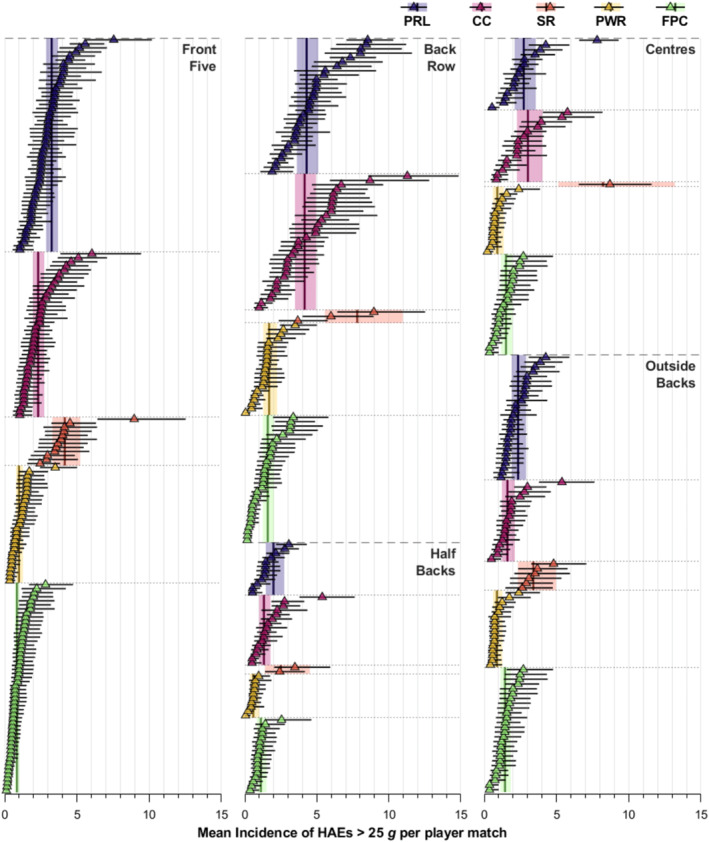
The mean incidence of HAEs > 25 g for each positional group and competition per median playing time in addition to each individual player's mean incidence. Only players who participated in three or more matches in the model were included for figure clarity.

Figure 6 illustrates the estimated mean incidence of HAEs > 25 g for each positional group and competition per median playing time in addition to each individual player's mean incidence. Within each positional group and competition, there was a wide range of HAE incidences between players, with certain players experiencing substantially more HAEs per FGE than the corresponding positional group mean incidence and their peers. For example, in Currie Cup, two back‐row players had a mean HAE incidence per FGE over twice that of the group's mean incidence (11.28 [8.57–14.85] and 8.69 [5.90–12.80] vs. 4.15 [3.47–4.95]). Similarly, a front‐five player in Farah Palmer Cup experienced approximately a three‐fold greater HAE incidence than the mean (2.83 [1.69–4.73] vs. 0.84 [0.70–1.01]), and a centre in Premiership Women's Rugby had an HAE incidence 2.7‐fold greater (2.39 [1.47–3.86) vs. 0.90 [0.60–1.35]) than their respective competition positional group mean.

## 4 Discussion

The aims of the present study were to quantify and compare the mean HAE incidence of players in and between different men's and women's rugby union competitions by the positional group; to quantify the incidence of HAEs across all contact events and to explore how incidence varies between players in the same position. An important finding of the present study was that some players experienced a substantially higher mean HAE incidence in comparison to the mean and other players within their respective competition and positional group, whereas other players experienced significantly fewer HAEs. This emphasises the potential importance of HAE mitigation strategies that individualise player‐monitoring processes and strategies to manage those whose exposure is deemed to be high. This argues against generic and potentially inappropriate management strategies that impose specific limits on players, since the individual variation means a single limit will both under and over‐manage players within a team, even when in the same playing position.

Furthermore, on average, men's players had a significantly higher HAE incidence per FGE in most positional groups and HAE magnitude thresholds when compared to women's players. Incidence of HAEs per FGE was lowest in scrums and highest for tackles and ball‐carries in both women's and men's players, whereas the maul was a relatively frequent source of HAEs for men's back row players. No significant differences were observed between competitions for the majority of playing positions and HAE magnitude thresholds per FGE in both men's and women's.

### 4.1 Individual Variation

This study makes a novel and important contribution to the HAE literature because it shows large individual player HAE differences compared to the positional group mean in each competition (Figure 6). Certain players experienced a higher mean incidence in comparison to the majority of other players. Due to natural variability around the mean, it is expected that some players will have more or fewer HAEs than the group mean. However, given that HAE exposure has implications for player welfare, particularly in the longer term (Daneshvar et al. 2023; Montenigro et al. 2017), research is required to explore potential reasons why some players experience more head accelerations than others (e.g., tackling and ball‐carrying technique (Woodward et al. 2024)) to help inform HAE mitigation interventions.

Based on the findings presented here, policymakers may wish to consider HAE mitigation strategies in rugby union that prioritise individual player monitoring processes and management strategies for those whose exposure is deemed to be excessive. However, in the present study, only one season of data were collected. Therefore, it is not possible to know if players naturally vary high or low relative to the mean across different seasons and thus whether a single season's HAE exposure is representative of their whole career. Research is urgently needed to understand both the cause and clinical relevance of cumulative HAE exposure over multiple seasons and whole careers in rugby union players to help guide such mitigation strategies.

### 4.2 HAE Incidence in Women's Versus Men's Competitions

For a large proportion of playing positions and magnitude thresholds, mean HAE incidence per FGE was significantly greater for men's players than women's players except for PAA > 4 krad/s^2^ and PLA > 55 g and PAA > 4 krad/s^2^ (Figure 1). Similar observations were made in a sample of elite players from different competitions (Tooby et al. 2023), in which men were reported to have significantly higher mean HAE incidence per 60 min of match play than women at ‘medium’ (PLA between 5 and 30 g and PAA between 400 and 2.0 krad/s^2^) and ‘higher’ magnitudes (PLA ≥ 30 g or PAA ≥ 2.0 krad/s^2^). Given that the frequency of collision events in matches is similar between the sexes (Roe, Sawczuk, Collins, et al. 2024), this finding suggests that intensity of contact events is greater in the men's game. This is potentially due to dissimilarities in physical characteristics between male and female rugby union players (Posthumus et al. 2020; Yao et al. 2021) and thus the potential forces that can be produced and experienced during contact‐events.

At the highest magnitude thresholds modelled in the present study (PLA > 55 g, PAA > 4 krad/s^2^ or a combination of both), differences between women's and men's players were all < 0.4 HAE per FGE and/or not significant (Figure 1). However, it is likely that these differences would broaden and become statistically and practically significant across multiple games and seasons (e.g., half or full season and playing career) (Sawczuk et al. 2024). Higher HAE magnitudes have been associated with significant changes in brain biomarkers (> 50 g Bari et al. 2019; Svaldi et al. 2020) and increased concussion risk (increases from 50% upwards starting at magnitudes approximately > 60 g in American Football (Freeman 2018)). In the present study, the incidence of higher magnitude HAEs for PLA (> 55 g) was significantly greater in the men's than women's game for most positions (Figure 1). However, recent data from high‐level domestic competition in England suggests that concussion incidence is similar between the sexes (Williams 2024). Therefore, it is possible that women players are more susceptible to concussions at lower magnitudes of HAE in comparison to men. Further research is required to establish the relationship between HAEs and concussion in both sexes and the implications for long‐term brain health outcomes.

### 4.3 HAE Incidence in Contact‐Events

Similar to previous research, tackle and ball‐carry had the highest incidence (Roe, Sawczuk, Owen, et al. 2024; Tooby et al. 2023), whereas a novel finding of the study was that scrums had the lowest incidence. For example, on average, a front‐five player would be expected to experience one HAE > 25 g in approximately 209 FGEs for women's and 25 FGE's for men's players from scrums, respectively, whereas back row players would be expected to experience one HAE > 25 g from scrums in every 115 and 31 FGE's for men's and women's players respectively. This is comparatively much lower than the documented one HAE (> 25 g) every 2.2 and 0.9 FGEs for tackles in women's and men's players, respectively. Furthermore, another novel finding was that mauls may provide a frequent source of HAE for men's back row players (e.g., one in every 1.1 FGE; PLA > 25 g), whereas lineouts were comparatively lower for all positions (approximately one in every five FGEs in men's and one in every 25 for women's players; PLA > 25 g).

### 4.4 HAE Incidence in Women's and Men's Competitions

There were no significant differences in HAE incidence rates between the women's competitions per positional group except for overall HAE incidence in outside backs (Figure 1). Similarly, in men's competitions, incidence rates of HAE were predominantly similar for each competition per positional group (Figure 1). These findings support recent research which reported similarities between different women's and men's competitions with respect to the number of contact‐events that players were involved in during a match (Roe, Sawczuk, Collins, et al. 2024). Where differences were observed between men's competitions, the majority were at lower magnitude thresholds (All HAEs, PLA > 25 g, PAA > 2 krad/s^2^ or a combination of both) with absolute differences ranging from approximately 1.5–5.4 HAE per FGE on average.

However, competitions may vary with respect to the number of games players play. For example, players in the Currie Cup may play a maximum of 14 games, whereas in Premiership Rugby, players may play up to 20 matches in a competitive season. Furthermore, players also typically play in multiple competitions throughout a season (e.g., domestic league and cup, in addition to international), so the actual number of matches a player could play may be in excess of 30 FGEs (Sawczuk et al. 2024). Thus, the total seasonal HAE exposure of players in different competitions, and players within these competitions is likely to vary within a season and across seasons (Sawczuk et al. 2024). Therefore, policymakers considering monitoring and possibly regulating HAE exposure may need to consider the total number of potential games played by a player in a competitive season or individual HAE limits, over shorter (e.g., months), medium (e.g., playing season) or longer‐term (e.g., players career) periods of time, within an individualised monitoring process as described above.

### 4.5 Limitations

Although this study provides several important and novel insights, it has some limitations. As in other iMG research (e.g., Tooby et al. 2023; Roe, Sawczuk, Owen, et al. 2024), recruitment of players relied on voluntary participation, which likely resulted in volunteer and sampling bias. Additionally, the analysis undertaken in the current study presented data per FGE to allow comparisons between competitions and sexes. As can be seen in Figure 1, even when confidence intervals overlapped, point estimates often differed. Thus, it is possible that these differences would have been magnified if multiple games were modelled (e.g., a half or full season). Furthermore, international competition was not assessed. Given that collision intensities increase as competition standard increases (Tierney et al. 2021), it is possible that the number of HAEs experienced by international players may be higher than domestic. Finally, the accuracy of the unmodelled incidence estimates is uncertain as they do not account for the hierarchical structure of the data. Attempts to model the structure of the data using Poisson and Binomial distributions were unsuccessful. Although unmodelled incidences are the norm in sports injury and HAE literature, future research should attempt to find modelling solutions for this problem so that more accurate estimates of incidence can be produced.

## 5 Conclusion

This study provides HAE incidences across a range of magnitude thresholds in men's and women's top domestic competitions per positional group contact‐event and at an individual level. The results demonstrated that even within positional groups, there was a high between‐player variability, with some players experiencing up to three times greater mean HAE incidence than their positional average. This emphasises the importance of HAE mitigation strategies that prioritise individual player monitoring processes and management strategies. Differences in HAE incidence between men's and women's competition per FGE suggest that women may be more susceptible to concussion at lower HAE magnitudes in comparison to men. However, future longitudinal research is required to investigate these relationships. In addition to the tackle and ruck, mauls may significantly contribute to HAE exposure in men's back row, whereas the incidence of HAEs in scrums and lineouts is of less concern for both men's and women's players. Although HAE incidence within men's and women's competitions were similar per FGE, differences in competition lengths will influence players' seasonal HAE exposure.

## Conflicts of Interest

The authors declare no conflicts of interest.

## References

[ejsc12295-bib-0001] Bari, S. , D. O. Svaldi , I. Jang , et al. 2019. “Dependence on Subconcussive Impacts of Brain Metabolism in Collision Sport Athletes: An MR Spectroscopic Study.” Brain Imaging and Behavior 13, no. 3: 735–749. 10.1007/s11682-018-9861-9. 29802602

[ejsc12295-bib-0002] Brooks, M. E. , B. Bolker , K. Kristensen , et al. 2024. glmmTMB: Generalized Linear Mixed Models Using Template Model Builder: Retrieved from 01/04/2024.

[ejsc12295-bib-0003] Bussey, M. D. , D. Salmon , J. Romanchuk , et al. 2023. “Head Acceleration Events in Male Community Rugby Players: An Observational Cohort Study Across Four Playing Grades, From Under‐13 to Senior Men.” Sports Medicine 54, no. 2: 517–530. 10.1007/s40279-023-01923-z. 37676621 PMC10933157

[ejsc12295-bib-0004] Bussey, M. D. , D. Salmon , J. Romanchuk , et al. 2024. “Head Acceleration Events in Male Community Rugby Players: An Observational Cohort Study Across Four Playing Grades, From Under‐13 to Senior Men.” Sports Medicine 54, no. 2: 517–530. 10.1007/s40279-023-01923-z. 37676621 PMC10933157

[ejsc12295-bib-0005] Daneshvar, D. H. , E. S. Nair , Z. H. Baucom , et al. 2023. “Leveraging Football Accelerometer Data to Quantify Associations Between Repetitive Head Impacts and Chronic Traumatic Encephalopathy in Males.” Nature Communications 14, no. 1: 3470. 10.1038/s41467-023-39183-0. PMC10281995 37340004

[ejsc12295-bib-0006] Freeman, M. D. 2018. “Concussion Risk From Helmeted Sports; A Re‐examination of Data and Methods.” Journal of Forensic Biomechanics 9, no. 1. 10.4172/2090-2697.1000139.

[ejsc12295-bib-0007] Heyward, O. , S. Emmonds , G. Roe , S. Scantlebury , K. Stokes , and B. Jones . 2022. “Applied Sports Science and Sports Medicine in Women's Rugby: Systematic Scoping Review and Delphi Study to Establish Future Research Priorities.” BMJ Open Sport & Exercise Medicine 8, no. 3: e001287. 10.1136/bmjsem-2021-001287. PMC9310180 35979431

[ejsc12295-bib-0008] Iverson, G. L. , R. J. Castellani , J. D. Cassidy , et al. 2023. “Examining Later‐In‐Life Health Risks Associated With Sport‐Related Concussion and Repetitive Head Impacts: A Systematic Review of Case‐Control and Cohort Studies.” British Journal of Sports Medicine 57, no. 12: 810–824. 10.1136/bjsports-2023-106890. 37316187

[ejsc12295-bib-0009] Jones, B. , J. Tooby , D. Weaving , et al. 2022. “Ready for Impact? A Validity and Feasibility Study of Instrumented Mouthguards (iMGs).” British Journal of Sports Medicine 56, no. 20: 1171–1179. 10.1136/bjsports-2022-105523. 35879022

[ejsc12295-bib-0010] Kuo, C. , D. Patton , T. Rooks , et al. 2022. “On‐Field Deployment and Validation for Wearable Devices.” Annals of Biomedical Engineering 50, no. 11: 1372–1388. 10.1007/s10439-022-03001-3. 35960418 PMC9652208

[ejsc12295-bib-0011] Lenth, R. , B. M. Bolker , P. Buerkner , et al. 2024. Emmeans: Estimated Marginal Means, Aka Least‐Squares Means: Retrieved 01/04/2024, from.

[ejsc12295-bib-0012] Montenigro, P. H. , M. L. Alosco , B. M. Martin , et al. 2017. “Cumulative Head Impact Exposure Predicts Later‐Life Depression, Apathy, Executive Dysfunction, and Cognitive Impairment in Former High School and College Football Players.” Journal of Neurotrauma 34, no. 2: 328–340. 10.1089/neu.2016.4413. 27029716 PMC5220530

[ejsc12295-bib-0013] Noguchi, K. , and F. Marmolejo‐Ramos . 2016. “Assessing Equality of Means Using the Overlap of Range‐Preserving Confidence Intervals.” American Statistician 70, no. 4: 325–334. 10.1080/00031305.2016.1200487.

[ejsc12295-bib-0014] Posthumus, L. , C. Macgregor , P. Winwood , K. Darry , M. Driller , and N. Gill . 2020. “Physical and Fitness Characteristics of Elite Professional Rugby Union Players.” Sports (Basel) 8, no. 6: 85. 10.3390/sports8060085. 32517080 PMC7353640

[ejsc12295-bib-0015] Quarrie, K. L. , W. G. Hopkins , M. J. Anthony , and N. D. Gill . 2013. “Positional Demands of International Rugby Union: Evaluation of Player Actions and Movements.” Journal of Science and Medicine in Sport 16, no. 4: 353–359. 10.1016/j.jsams.2012.08.005. 22975233

[ejsc12295-bib-0016] Roe, G. , T. Sawczuk , N. Collins , et al. 2024. “Spot the Difference? Contact‐Event Frequency During >30,000 Women’s and Men’s Rugby Union Player Matches, Across Top Domestic and International Competitions.” European Journal of Sport Science, Under Review. 10.1002/ejsc.12307 PMC12009008 40252212

[ejsc12295-bib-0017] Roe, G. , T. Sawczuk , C. Owen , et al. 2024. “Head Acceleration Events During Tackle, Ball‐Carry, and Ruck Events in Professional Southern Hemisphere Men's Rugby Union Matches: A Study Using Instrumented Mouthguards.” Scandinavian Journal of Medicine & Science in Sports 34, no. 6: e14676. 10.1111/sms.14676. 38867444

[ejsc12295-bib-0018] Rothman, K. J. 2012. Epidemiology: An Introduction. Oxford University Press.

[ejsc12295-bib-0019] Sawczuk, T. , M. Cross , C. Owen , et al. 2024. “The Application of Match‐Event and Instrumented Mouthguard (iMG) Data to Inform Match Limits: An Example Using Rugby Union Premiership and Rugby League Super League Data From England.” European Journal of Sport Science, Ahead of Print. 10.1002/ejsc.12188 PMC11534626 39305464

[ejsc12295-bib-0020] Speelman, C. P. , and M. McGann . 2013. “How Mean Is the Mean?” Frontiers in Psychology 4: 451. 10.3389/fpsyg.2013.00451. 23888147 PMC3719041

[ejsc12295-bib-0021] Stevenson, M. , and M. Sergeant . 2024. Tools for the Analysis of Epidemiological Data.

[ejsc12295-bib-0022] Svaldi, D. O. , C. Joshi , E. C. McCuen , et al. 2020. “Accumulation of High Magnitude Acceleration Events Predicts Cerebrovascular Reactivity Changes in Female High School Soccer Athletes.” Brain Imaging Behav 14, no. 1: 164–174. 10.1007/s11682-018-9983-0. 30377933

[ejsc12295-bib-0023] Tierney, P. , C. Blake , and E. Delahunt . 2021. “Physical Characteristics of Different Professional Rugby Union Competition Levels.” Journal of Science and Medicine in Sport 24, no. 12: 1267–1271. 10.1016/j.jsams.2021.05.009. 34144858

[ejsc12295-bib-0024] Tooby, J. , K. Till , A. Gardner , et al. 2024. “When to Pull the Trigger: Conceptual Considerations for Approximating Head Acceleration Events Using Instrumented Mouthguards.” Sports Medicine 54, no. 6: 1361–1369. 10.1007/s40279-024-02012-5. 38460080 PMC11239719

[ejsc12295-bib-0025] Tooby, J. , J. Woodward , R. Tucker , et al. 2023. “Instrumented Mouthguards in Elite‐Level Men’s and Women’s Rugby Union: The Incidence and Propensity of Head Acceleration Events in Matches.” Sports Medicine 54, no. 5: 1327–1338. 10.1007/s40279-023-01953-7. 37906425 PMC11127838

[ejsc12295-bib-0026] Wang, T. , R. Kenny , and L. C. Wu . 2021. “Head Impact Sensor Triggering Bias Introduced by Linear Acceleration Thresholding.” Annals of Biomedical Engineering 49, no. 12: 3189–3199. 10.1007/s10439-021-02868-y. 34622314

[ejsc12295-bib-0027] Williams, S. 2024. WRISP Report 2023‐2024: Retrieved 23/09/2024, from.

[ejsc12295-bib-0028] Woodward, J. , J. Tooby , R. Tucker , et al. 2024. “Instrumented Mouthguards in Elite‐Level Men's and Women's Rugby Union: Characterising Tackle‐Based Head Acceleration Events.” BMJ Open Sport & Exercise Medicine 10, no. 3: e002013. 10.1136/bmjsem-2024-002013. PMC11298745 39104376

[ejsc12295-bib-0029] Yao, X. , C. Curtis , A. Turner , C. Bishop , A. Austerberry , and S. Chavda . 2021. “Anthropometric Profiles and Physical Characteristics in Competitive Female English Premiership Rugby Union Players.” International Journal of Sports Physiology and Performance 16, no. 9: 1234–1241. 10.1123/ijspp.2020-0017. 33626507

